# Predicting the spatio-temporal infection risk in indoor spaces using an efficient airborne transmission model

**DOI:** 10.1098/rspa.2021.0383

**Published:** 2022-03

**Authors:** Zechariah Lau, Ian M. Griffiths, Aaron English, Katerina Kaouri

**Affiliations:** ^1^ School of Mathematics, Cardiff University, CF24 4AG Cardiff, UK; ^2^ Mathematical Institute, University of Oxford, OX1 6GG Oxford, UK; ^3^ Department of Mechanical, Aerospace and Civil Engineering, University of Manchester, M13 9PL Manchester, UK

**Keywords:** COVID-19, airborne transmission, infection risk, modelling, ventilation

## Abstract

We develop a spatially dependent generalization to the Wells–Riley model, which determines the infection risk due to airborne transmission of viruses. We assume that the infectious aerosol concentration is governed by an advection–diffusion–reaction equation with the aerosols advected by airflow, diffused due to turbulence, emitted by infected people, and removed due to ventilation, inactivation of the virus and gravitational settling. We consider one asymptomatic or presymptomatic infectious person breathing or talking, with or without a mask, and model a quasi-three-dimensional set-up that incorporates a recirculating air-conditioning flow. We derive a semi-analytic solution that enables fast simulations and compare our predictions to three real-life case studies—a courtroom, a restaurant, and a hospital ward—demonstrating good agreement. We then generate predictions for the concentration and the infection risk in a classroom, for four different ventilation settings. We quantify the significant reduction in the concentration and the infection risk as ventilation improves, and derive appropriate power laws. The model can be easily updated for different parameter values and can be used to make predictions on the expected time taken to become infected, for any location, emission rate, and ventilation level. The results have direct applicability in mitigating the spread of the COVID-19 pandemic.

## 1. Introduction

The COVID-19 pandemic has spread rapidly across the globe, with more than 245 million confirmed cases worldwide and almost five million deaths, at the time of writing [1]. The virus causing the pandemic, SARS-CoV-2, is transmitted through virus-carrying respiratory aerosols, which are released when an infected person coughs, sneezes, talks or breathes [2,3]. Evidence has accumulated that airborne aerosols formed from smaller respiratory droplets evaporating can also transmit the disease [4]. In July 2020, 239 scientists signed an open letter appealing for the recognition of airborne transmission [5] and in October 2020 the US Centre for Disease Prevention and Control acknowledged airborne transmission and updated its guidelines [6]. In March 2021, Public Health England recommended improving ventilation of indoor spaces as an intervention against COVID-19 [7].

Models studying the risk of airborne transmission generally fall into one of two types: Computational Fluid Dynamics (CFD) models, and the Wells–Riley model and its extensions. Markov-chain methods have also been used to study airborne transmission, in global population scenarios [8–11] and in local space-dependent scenarios [9,10,12,13]. CFD models are useful in studying airborne transmission as they take into account the room size, geometry, complex turbulent airflow, and size distribution of the aerosols. Various CFD models have been applied to the COVID-19 pandemic to investigate the transport of aerosols [14–19]. Some of these studies focus on transport in the short term (less than 5 min) [14–16], while others show the build-up of aerosols indoors over an hour [18]. The risk of infection can then be estimated from these results [18,19]. However, CFD models require specialized software and are computationally demanding. They take a long time to run even for small locations, so they cannot easily be applied to a new location and are hence not suited for ‘dynamic simulations’, which are essential in a fast-evolving pandemic.

On the other hand, the Wells–Riley model [20,21] and its generalizations such as the Gammaitoni–Nucci extension [22] are built on the Well-Mixed-Room (WMR) assumption, which assumes that the virus-carrying aerosols are instantaneously evenly distributed throughout the room [20–22]. This assumption implies that everyone in the room is equally likely to be infected, regardless of their position. This is a major simplification of the problem as it neglects the complex effects of airflow on the aerosols and the effects of the room geometry. However, Wells–Riley models are easy to implement and very fast to run [22,23], and they have been extensively applied to make predictions about COVID-19 transmission [23–29].

In this paper, we determine the *spatially dependent* viral concentration and the airborne infection risk in a room through developing an extension to the Wells–Riley model [20,22]. The model describes the concentration of aerosols via an advection–diffusion–reaction equation and allows fast simulations while still taking into account the effect of turbulent airflow. Although this model is more complex than the Wells–Riley model, it is significantly less computationally expensive than CFD models. Consequently, the model sufficiently reproduces results of CFD simulations while running quickly on an average PC. The computational simplicity of our model is an asset during the fast decision-making that is required in a fast-evolving pandemic as it enables quick, easy application to different locations such as classrooms, healthcare clinics, and restaurants.

In §2, we present our model. We assume a single presymptomatic or asymptomatic spreader of COVID-19 who is breathing or talking in a room with only mechanical ventilation (no open doors or windows). We model the recirculation of the air due to air-conditioning by modelling the concentration at all positions on a loop around the room, which provides a quasi-three-dimensional set-up. In this case, we show that a semi-analytic solution exists for the concentration as a function of space and time. We then use the concentration to determine the infection risk, using the Wells–Riley ansatz. In §3, we discuss the parameter values in our model and how these may be easily changed once updated knowledge becomes available.

In §4, we apply our model to three real-life case studies and find good agreement with data. We first compare the model with data from two superspreader outbreaks. The first outbreak occurred at a courtroom in Switzerland in October 2020 [30], where five out of 10 people present tested positive a few days after a hearing. The second outbreak occurred at a restaurant in Guangzhou, China, in January 2020; seven to nine people out of 20 were infected [31]. Satisfactory agreement with two CFD models that simulated the restaurant outbreak is also obtained [17,32]. We also consider air-sampling data in a hospital ward, as reported in [33]. Subsequently, in §5 we apply the model to an average-sized classroom. We determine how the aerosol concentration and the infection risk change for four different ventilation settings: very poor ventilation, poor ventilation, the ASHRAE (American Society of Heating, Refrigeration and Air-Conditioning Engineers [81]) recommended minimum ventilation for classrooms, and a ventilation setting better than the ASHRAE recommended minimum.

In §6, we summarize our work and indicate how policy-makers and other decision-makers could use the model in future planning to tackle the pandemic.

## 2. Modelling framework

### (a) The advection–diffusion–reaction equation

**Figure 1.**
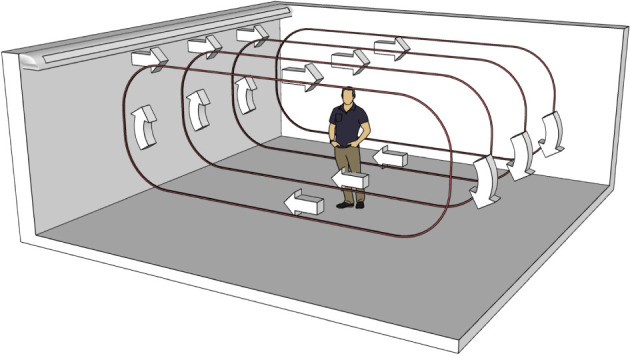
Schematic of the room configuration illustrating the assumption of air recirculation in the room generated by an air-conditioning unit. (Online version in colour.)

Below, we develop our model and explain major assumptions. As discussed in the Introduction, while the Wells–Riley model leads to fast predictions, it does not capture spatial effects. Since a CFD approach is very time consuming, we develop a quick-to-run quasi-three-dimensional, spatio-temporal model for the concentration of viral aerosols indoors and the infection risk. We consider an infectious person talking or breathing, with or without a mask. We also assume that the room contains an air-conditioning unit, which drives a recirculating flow, as shown in figure 1 and as predicted by van Hooff *et al*. [34]. We show that the model possesses a semi-analytic solution and, thus, predictions can be generated very quickly.

The infectious person acts as a source of infectious aerosols, $S_{\text{inf}}$. For simplicity, we make the assumption that all aerosols are of the same size and carry the same amount of virus, but note that this can be readily generalized for particle-size distributions [35]. The aerosols are transported by advection caused by the airflow, which follows the recirculating loop as shown in figure 1, and has velocity, v. Even though air is expelled from the mouth and nose during breathing and talking, this is a local and temporary effect, so it is fair to assume that the aerosols are passively released into the flow stream,^1^ so that the advection velocity of the aerosols is also v.

The infectious aerosols are assumed to be removed due to three factors: ventilation, biological deactivation of the virus, and gravitational settling. These correspond to sink terms, which we denote by $S_{\text{vent}}$, $S_{\text{deact}}$, and $S_{\text{set}}$, respectively.

Finally, we assume turbulent mixing of the air. Turbulence leads to the aerosols diffusing much more rapidly than due to Brownian motion [36]. Turbulent diffusion is governed by the eddy diffusion coefficient, $K\;(m^{2}/s)$ [36]. More details are given in §2(b).

**Figure 2.**
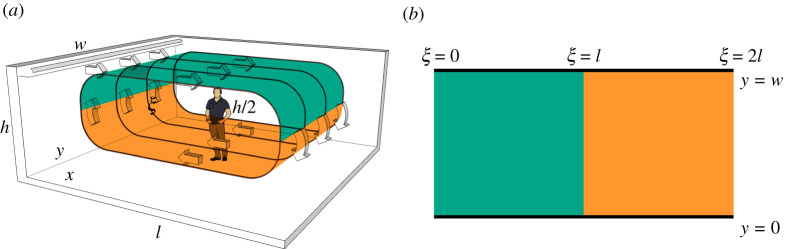
(*a*) Quasi-three-dimensional set-up with air recirculating in a room of dimensions l×w×h; ξ denotes the arclength around the loop surface of the recirculating flow. We divide the loop into an upper (blue/darker) and lower (green/lighter) domain; x and y denote Cartesian coordinates that describe the position on the floor. The upper half of the loop is projected onto the Cartesian plane via x=ξ, 0≤ξ≤l; the lower half is connected via x=2l−ξ, l≤ξ≤2l. The upper and lower branches of the surface are assumed to be separated by a distance h/2. (*b*) The unwrapping of the loop surface leads to a two-dimensional domain. (Online version in colour.)

From these assumptions, we arrive at the advection–diffusion–reaction (ADR) equation, the governing equation for the concentration of infectious aerosols,

2.1 $$\frac{\partial C}{\partial t}+\nabla \cdot (vC)-\nabla \cdot (K\nabla C)=S_{\text{inf}}-S_{\text{vent}}-S_{\text{deact}}-S_{\text{set}},$$

where C is the concentration of infectious aerosols (aerosols/m${\;}^{2}$) at all points on the surface of the looping airflow, t is the time (s) [37], and ∇=(∂/∂ξ,∂/∂y) is the two-dimensional gradient operator in the surface of the looping airflow, where ξ (m) denotes the arclength in the streamwise direction of the flow and y (m) is the coordinate transverse to the flow. The upper and lower branches of the surface are assumed to be separated by a distance h/2, following [34] (figure 2*a*).

### (b) Further modelling assumptions: a simplified advection–diffusion–reaction equation

Following [38], we model an infected person who is breathing or talking as a continuous point source emitting virus-carrying aerosols at a constant rate of R aerosols/s. We choose to use the infectious aerosol emission rate instead of the traditional quanta generation rate in the Wells–Riley model for reasons that are discussed in §2(d). We also assume that inhaling and exhaling occur at the same rate, so there is no net source or sink of air from the emitter and receiver of the aerosols. Thus, an infectious person talking or breathing at position ($\xi_{0}$,$y_{0}$) is modelled as follows:

2.2 $$S_{\text{inf}}=R\delta (\xi -\xi_{0})\delta (y-y_{0}),$$

where δ(x) is the Dirac delta function.

Furthermore, we assume there is mechanical ventilation provided by air-conditioning units. Following [22,24,39–41], we model the ventilation effect as a sink term of uniform strength over the domain

2.3 $$S_{\text{vent}}=-\lambda C,$$

where λ is the air exchange rate of the room, measured in $s^{-1}$. This is an approximation, since ventilation is a localized effect, with removal of the air occurring at a higher rate near the air vents and openings in the room [40,42]. However, our approach still allows inclusion of the effect of ventilation while retaining its key features and without greatly increasing the mathematical complexity of our model.

Following [22], we model the biological deactivation and gravitational settling as global first-order removals, as follows:

2.4 $$S_{\text{deact}}=-\beta C$$

and

2.5 $$S_{\text{set}}=-\sigma C,$$

where β is the viral deactivation rate and σ is the aerosol settling rate, both measured in $s^{-1}$. While gravitational settling would induce interaction between the two layers due to the size of the aerosols (about 5 µm [2]), it would take about 4 h for them to fall from layer 1 to layer 2, if a perfect vertical fall is assumed. However, these aerosols will not follow a perfect vertical motion as a result of the recirculation; it takes only minutes for an aerosol to loop around the room. Thus, the aerosols are much more likely to be carried from the upper layer to the lower layer by the recirculating airflow than fall into the lower layer from the upper layer. Furthermore, most of the virus in the aerosols is deactivated within 1–2 h (see §2(a)). Therefore, in our model we assume that we can neglect the layer–layer interaction.

The typical airflow from an air-conditioning unit, $|v|=0.1-1\;{m\;s}^{-1}$ [43], is small compared with the speed of sound ($340\;{m\;s}^{-1}$), so we may assume that the air is an incompressible fluid [44]; that is,

2.6 ∇⋅v=0.

The velocity, v, is controlled solely by the air-conditioning unit, which we assume has an inlet in the left wall, and we assume that the airflow velocity at all points in the surface of the loop is constant and uniform, i.e. v=(v,0), where v is a constant.

To determine K, the eddy diffusion coefficient, we use the following formula:

2.7 $$K=c_{k}Q{\left(2c_{\epsilon }VN^{2}\right)}^{-1/3}.$$

Here, $c_{k}$ is the von Karman constant, Q is the total volume flow rate into the room, V is the room volume, N is the number of air supply vents, and $c_{\epsilon }$ is the constant of proportionality in Taylor’s Dissipation Law [45,46]. The relation (2.7) was presented in [41] and is based on the turbulent kinetic energy balance (TKEB) relationship initially proposed in [45] for rooms served by mixing ventilation (i.e. when cool air is blown in through the ceiling or wall and dilutes the room air [47]; see figure 1 for an example). Since Q=Vλ, (2.7) may be rewritten as

2.8 $$K=c_{k}V\lambda {\left(2c_{\epsilon }VN^{2}\right)}^{-1/3}.$$

We assume the relationship $c_{\epsilon }=c_{k}^{3}$ following [45,48]. Taking $c_{k}=0.39$ [49] gives $c_{\epsilon }=0.059$. (We note that different formulae for $c_{\epsilon }$ have been used, for example, $c_{\epsilon }=c_{k}$ [39] and $c_{\epsilon }=0.5c_{k}^{3}$ [41], albeit without providing a rationale.)

For a room with length l and width w, we unwrap the loop surface of the airflow to the two-dimensional domain (ξ,y)∈[0,2l]×[0,w] (figure 2*b*). This extended domain allows us to model the evolution of the aerosol cloud in both the upper and lower layers of the flow stream in a simpler way. Assuming there is only one infectious person, we substitute equations (2.2)–(2.6) into the ADR equation (2.1) to obtain the partial differential equation (PDE) we will be solving, as follows:

2.9 $$\frac{\partial C}{\partial t}+v\frac{\partial C}{\partial \xi }-K\left(\frac{\partial^{2}C}{\partial \xi^{2}}+\frac{\partial^{2}C}{\partial y^{2}}\right)=R\delta (\xi -\xi_{0})\delta (y-y_{0})-(\lambda +\beta +\sigma )C.$$

We assume that no virus-carrying aerosols are present initially. Hence, the initial condition is

2.10 C(ξ,y,0)=0.

We model the recirculating flow using the following domain and boundary conditions. In our set-up, the aerosols loop around the room; we model this through periodic boundary conditions on the concentration and its derivative at the wall ξ=0 and opposite side of the extended domain, at ξ=2l. Hence,

2.11a C(0,y,t)=C(2l,y,t)

and

2.11b $$\frac{\partial C}{\partial \xi }(0,y,t)=\frac{\partial C}{\partial \xi }(2l,y,t).$$

The system is closed by applying Neumann boundary conditions at the walls located at y=0,w as follows:

2.11c $$\frac{\partial C}{\partial y}(\xi ,0,t)=\frac{\partial C}{\partial y}(\xi ,w,t)=0.$$

The conditions (2.11*c*) correspond to perfect reflection of aerosols at the wall. In practice, some aerosols may be absorbed by the wall and so our model represents a worst-case scenario in this respect.

### (c) The semi-analytic solution for the viral aerosol concentration

We solve the PDE (2.9) with the boundary conditions (2.11*a*–*c*) and initial condition (2.10) by first solving the homogeneous problem to determine the impulse function. Then, we convolve the impulse with the source function (2.2) to obtain the full solution (see [50] for details). A solution to the homogeneous problem of the form $C(\xi ,y,t)=e^{-(\lambda +\beta +\sigma )t}A(\xi ,y,t)$ is assumed [50]. We then use separation of variables to reduce the problem to two one-dimensional diffusion problems [50]. Owing to the periodic and Neumann boundary conditions (2.11*a*–*c*), we can use the method of images [51] when solving for the impulse function. Hence, the solution is given by

2.12 $$\begin{array}{rl} C(\xi ,y,t) & \;=\int_{0}^{t}\frac{R}{4\pi K\tau }e^{-(\lambda +\beta +\sigma )\tau }\sum_{m=-\infty }^{\infty }e^{-({\left(\xi -v\tau -\xi_{0}-2ml\right)}^{2}/4K\tau )}\\ & \;\;\times \sum_{n=-\infty }^{\infty }(e^{-({\left(y-y_{0}-2nw\right)}^{2}/4K\tau )}+e^{-({\left(y+y_{0}-2nw\right)}^{2}/4K\tau )})d\tau . \end{array}$$

Now, by leveraging the mapping of the quasi-three-dimensional set-up to a two-dimensional geometry, we can average the aerosol concentration in the upper and lower branches of the loop surface to determine the concentration for the quasi-three-dimensional set-up. Specifically, we define x∈[0,l] to be the Cartesian coordinate in the direction of the upper flow, so x=ξ, 0≤ξ≤l defines the upper branch and x=2l−ξ defines the lower branch, for l≤ξ≤2l (figure 2*a*). The concentration of viral aerosols in the upper branch is then given by

2.13 $$C_{\text{upper}}(x,y,t)=C(x,y,t),$$

and the concentration of viral aerosols in the lower branch is given by

2.14 $$C_{\text{lower}}(x,y,t)=C(2l-x,y,t).$$

Recall that we assume the separation between the two branches is half the height of the room. We assume that within the two branches the air is well mixed and use this to transform the two-dimensional expressions back into a three-dimensional representation

2.15 $$C(x,y,t)=\frac{\left(C_{\text{upper}}(x,y,t)+C_{\text{lower}}(x,y,t)\right)}{h/2}.$$

By modifying equation (2.15) and placing the source in the appropriate layer, we are equally able to account for people who are standing, sitting, or lying down. We then substitute (2.12) into (2.15) to obtain the total concentration

2.16 $$\begin{array}{rl} & C(x,y,t)=\frac{2R}{4\pi Kh}\int_{0}^{t}\sum_{m=-\infty }^{\infty }(e^{-({\left(x-v\tau -x_{0}-2ml\right)}^{2}/4K\tau )}+e^{-({\left(x+v\tau +x_{0}-2ml\right)}^{2}/4K\tau )}) \end{array}$$

2.17 $$\begin{array}{rl} & \;\;\times \sum_{n=-\infty }^{\infty }(e^{-({\left(y-y_{0}-2nw\right)}^{2}/4K\tau )}+e^{-({\left(y+y_{0}-2nw\right)}^{2}/4K\tau )})\frac{e^{-(\lambda +\beta +\sigma )\tau }}{\tau }\;d\tau . \end{array}$$

### (d) The probability of infection (infection risk)

Following the Wells–Riley ansatz, we assume an exponential probability density function for a susceptible person being infected from aerosols as a function of the dose of viral aerosols inhaled, d [20,29,52],

2.18 P(d)=1−exp(−Id).

We then calculate d from the formula given in [15,20] as

2.19 $$d(x,y,t)=\int_{0}^{t}\rho C(x,y,\tau )\;d\tau .$$

Here, ρ is the average breathing rate of the susceptible person, i.e. the average volume of air inhaled per second, and C(x,y,t) is given by (2.17). Equations (2.18) and (2.19) may be combined to give

2.20 $$P(x,y,t)=1-\text{exp}\left(-I\int_{0}^{t}\rho C(x,y,\tau )\;d\tau \right).$$

Here, I is a conversion factor that allows to obtain the number of infectious aerosols in terms of the quanta emission rate [24]. A quantum is defined as the dose of airborne virions corresponding to an infection risk of $1-e^{-1}\approx 63\%$; that is, 63% of susceptibles getting infected [20,21]. The parameter I is also related to the median infectious dose, $d_{m}$, the dose of airborne virions causing infection to 50% of the susceptible population, by

2.21 $$\frac{1}{2}=\left\{ \begin{array}{ll} 1-\exp(-Id_{m}) & \text{if}\;\mu \leq 1,\\ 1-\exp\left(-\frac{Id_{m}}{\mu }\right) & \text{if}\;\mu >1. \end{array}\right.$$

Here, μ is the expected number of viral copies in each aerosol, which is given by [35,53]

2.22 $$\mu =\frac{\pi }{6}d_{p}^{3}c_{v},$$

where $c_{v}$ is the viral load (viral copies/ml) of the virus in biological samples (usually sputum or saliva for SARS-CoV-2) and $d_{p}$ is the average diameter of the aerosols released. The quanta emission rate, q, is related to I via

2.23 q=RI.

We choose not to use the infectious quantum as the unit in (2.1) and (2.2), and to work in terms of R and I instead, since the predictions of this model may then be more easily regenerated when either of these parameter values is updated. The value of R changes with the type of activity but is currently uncertain. Also, the value of I may change independently since the dose may change with the severity of the COVID-19 disease [54,55]; age has also been suggested to influence disease severity [56,57]. Moreover, it has been suggested that some SARS-CoV-2 variants, such as the Delta variant, are more infectious corresponding to higher values of I [58].

## 3. Parameters

### (a) Parameter estimates

In this section, we discuss the model parameters that are independent of the room configuration and give the values we choose. For each case study we examine, additional location-specific parameter values are given in the electronic supplementary material.

#### (i) Infectious aerosol emission rate

The infectious aerosol emission rate, R, is obtained from the rate of the total number of aerosols generated, $R_{\text{total}}$, by

3.1 $$R=\left\{ \begin{array}{ll} \mu R_{\text{total}}, & \text{if }\mu <1,\\ R_{\text{total}}, & \text{if }\mu \geq 1. \end{array}\right.$$

Currently, there is no agreed value for the total aerosol emission rate, $R_{\text{total}}$, in the literature. Also, μ varies as it is proportional to the viral load, $c_{v}$, (see equation (2.22)), which varies for each infectious person and their stage of infection [59–61]. For instance, the airborne transmission models in [24,27,35,62] all use different values for $R_{\text{total}}$, following [63–66], respectively; these values are compiled in table 1. Some of these papers present the emission rate in terms of number of aerosols per volume of breath exhaled, $R_{V}=R_{\text{total}}/\rho_{\text{inf}}$, where $\rho_{\text{inf}}$ is the average breathing rate of the infectious person. However, there seems to be a consensus [63–65] that speaking is assumed to release significantly more aerosols than breathing; here, we assume that speaking generates 10 times more aerosols than breathing.

**Table 1.** The total aerosol emission rate, used in different models, for breathing and for talking (where available). For emission rates originally given in terms of aerosol per volume, $R_{V}$, we have used the conversion formula $R_{V}=R_{\mathrm{total}}/\rho_{\text{inf}}$, where the breathing rate, $\rho_{\text{inf}}=1.3\times 10^{-4}\;m^{3}\;s^{-1}$ [67]. We take the values from [64] for the analysis in this paper.

source	$R_{\mathrm{total}}$ (aerosols/s) for breathing	$R_{\mathrm{total}}$ (aerosols/s) for talking
[63]	13.1	42.9
[64]	8	80
[65]	9.2	20.5
[66]	—	1000

Following [27,64], we assume that the total aerosol emission rate, $R_{\text{total}}=8\;\mathrm{aerosols}/s$ for breathing and $R_{\text{total}}=80\;\mathrm{aerosols}/s$ for talking. The parameter values used are collected in table 2. We assume that the average aerosol diameter, $d_{m}=5$ µm according to the definition of airborne droplet nuclei from the WHO [2]. The viral load in the mouth, $c_{v}$, on the other hand, varies for each infectious person and their stage of infection [59–61]. Values of $c_{v}$ have been found in the range of $10^{3}$–$10^{12}$ viral copies ${\mathrm{ml}}^{-1}$, with the median value about $10^{6}$ viral copies ${\mathrm{ml}}^{-1}$ [59–61]. However, Srinivasan *et al*. [72] and Lee [68] report that the minimum viral loads required for aerosol transmission were $10^{8}$–$10^{9}$ viral copies ${\mathrm{ml}}^{-1}$. Hence, in our simulations for the two superspreader outbreaks (courtroom and restaurant), as well as for the study in the hypothetical classroom later, we choose $c_{v}\geq 10^{9}$ viral copies ${\mathrm{ml}}^{-1}$. Hence, from equation (2.22), μ≥6.5% of aerosols carry a copy of the SARS-CoV-2 virus. From equation (3.1), the emission rate of virus-carrying aerosols is then R≥0.5 aerosols/s for breathing and R≥5 aerosols/s for talking.

**Table 2.** Parameter estimates.

parameter	symbol	value	source
average breathing rate of susceptible person	ρ	$1.3\times 10^{-4}\;m^{3}\;s$	[67]
total aerosols emission rate	$R_{\text{total}}$	breathing: 8 aerosols/s	[64]
		talking: 80 aerosols/s	
viral load	$c_{v}$	$\geq 10^{9}$ viral copies/ml	[68]
average aerosol diameter	$d_{p}$	5 µm	[2]
viral copies per aerosol	μ	≥6.5% viral copies/aerosol	[53]
infectious aerosols emission rate	R	breathing: ≥0.5 aerosols/s	(3.1)
		talking: ≥5 aerosols/s	
efficiency of mask	η	0.5	[69]
virus deactivation rate	β	1.7$\times 10^{-4}\;s^{-1}$	[70]
gravitational settling rate	σ	1.1$\times 10^{-4}\;s^{-1}$	[35]
median infectious dose	$d_{m}$	100 virions	[15,71]
conversion factor to obtain dose in terms of infectious quantum	I	0.0069	(2.21)
quanta emission rate	q	breathing: 12.4 quanta/h	(2.23)
		talking: 124 quanta/h	

We note that in (2.17), C is proportional to the strength of the source, R, as expected. Henceforth, we will also use the dimensionless parameter $R=R/R_{0}$, where $R_{0}$ is the rate of emission of infectious aerosols when breathing (see tables 2 and 3). In this way, the results may be easily scaled when better estimates of R become available.

**Table 3.** The rate of emission of infectious aerosols by a person and the corresponding non-dimensional rate, R, when breathing or talking, and with and without a mask, scaled by the emission rate during breathing, $R_{0}=0.5$ aerosols/s.

infectious person state	R (aerosols/s)	$R=R/R_{0}$
breathing	0.5	1
talking	5	10
breathing with mask	0.25	0.5
talking with mask	2.5	5

#### (ii) Using a mask

We quantify the reduction in the infection risk when the infectious person wears a face mask. Therefore, we introduce a parameter, η, that measures the efficiency of the mask, so

3.2 $$R_{\text{mask}}=R(1-\eta ).$$

The efficiency of surgical masks and N95 respirators is well documented [73,74]. However, due to the sudden demand due to the COVID-19 pandemic, there has been a shortage of such masks and many people have had to manage with cloth masks. The efficiency of these masks is not well studied: at the moment there is only a preliminary study by Fischer *et al*. [69], which suggests that the efficiency is at least 50%. Furthermore, a fraction of people wear their masks incorrectly, which reduces their efficiency. Thus, we shall assume that a mask with an efficiency of 50% represents a reasonably realistic scenario.

#### (iii) Viral deactivation and gravitational settling

For the viral deactivation rate, β, according to [70], artificially generated aerosols carrying SARS-CoV-2 had a half-life of 1.1–1.2 h, which suggests that β is in the range of $0.58-0.63\;h^{-1}\approx 1.6-1.8\times 10^{-4}\;s^{-1}$. In our simulations, we will take $\beta =1.7\times 10^{-4}\;s^{-1}$. We must caution that this value of β is for artificially generated aerosols in a controlled laboratory environment and may be different for naturally produced bio-aerosols. However, our model can easily be updated if more accurate estimates emerge.

For the gravitational settling parameter, σ, we refer to [35]. Here, aerosols released by respiratory activities were found to have a mean value of $\sigma =0.39\;h^{-1}\approx 1.1\times 10^{-4}\;s^{-1}$; we take this value for our work.

#### (iv) Dose and infectious quantum

At present, the median infectious dose, $d_{m}$, is unknown for SARS-CoV-2. Early estimates by Vuorinen *et al*. [15], Basu [71], and Karimzadeh *et al*. [75] suggest that $d_{m}$ for airborne COVID-19 is O(100) virions. We take $d_{m}=100$ virions for our analysis but keep in mind that this may be updated in the future. Dabisch *et al*. [54] recently showed that in primates, $d_{m}$ for seroconversion was 52 and $d_{m}$ for fever was 256 virions; our choice lies between these values. As μ<1, when $d_{m}=100$ virions, (2.21) gives I≈0.0069.

Using the latter value of I and also R as in table 3, equation (2.23) gives q=12.4 quanta/h for breathing and 124 quanta/h for talking. In [25], they estimate the average q to be 14–48 quanta/h, which is comparable to our value for breathing. On the other hand, our values are in the 95th–99th percentile of the values obtained in [62]. Our high values of q are due to a higher value of $c_{v}$ that we assumed following [53,68] and because we chose a value of $d_{m}$ that probably corresponds to a worst-case scenario.

We collect parameter values for this section in table 2. The results of our model, as well as those of most other models for COVID-19 transmission, should be interpreted with caution until these values are confirmed.

### (b) Low-cost numerical implementation

In the subsequent simulations, we implement the solution for the concentration (2.17) in Python 3.8.5 64-bit. The convolutions are performed using the *convolve* function from the scipy.signal subpackage, which convolves two N-dimensional arrays [76]. To achieve satisfactory accuracy, we use a time step of 1s. The first infinite series in (2.17) is evaluated for m≤|vt/2l| as this is the number of times an aerosol travels around the recirculation surface during the time t. The second infinite series in (2.17) only needs to be evaluated for −3≤n≤3, as the steep exponential decay of the terms ensures very good accuracy with only the first few terms in the series [40]. For the courtroom case study (see §4(a)), as v=0, so m=n≤|3| (see electronic supplementary material, table S1 for more information). For the restaurant case study (see §4(b)), m≤|45|. For the hospital case study (see §4(c)), m≤|77|. Finally, for the hypothetical classroom case study (see §5), m≤135.

To determine the infection risk (2.19), we apply the *cumtrapz* function from the scipy.integrate subpackage, which cumulatively integrates an array of values using the trapezoidal rule [76], to the results of the convolutions. All plots are produced using the Matplotlib library [77].

The contour plots of the concentration and probability of infection are generated using a rectangular mesh of size 0.05 m. The computation time required is approximately 1 min for 1 h of real time. We run the simulations on a 2012 MacBook Pro laptop, with a 2.5GHz Dual Core Intel Core i5-3210M processor and 4GB 1805MHz RAM.

### (c) Ease of updating the model

Owing to our model’s quick runtime, the results can easily be updated when the values of R and I are updated; for example, due to new variants of the virus. We can also easily update our results for different settings, activity types, and ventilation levels. For example, we could quickly generate predictions for a person wearing a surgical mask or exercising in a gym. See electronic supplementary information, §1, for more details on how to update the results for several cases of interest.

## 4. Comparing the model with real-life case studies

We apply our model to a series of real-life case studies: two superspreader outbreaks in a courtroom in Switzerland [30] and at a restaurant in Guangzhou, China [31], and air-sampling data from a hospital ward [33]. Ventilation quality varies between these case studies; the courtroom was very poorly ventilated due to a breakdown of the ventilation system, the restaurant was poorly ventilated with recirculation of the air from the air-conditioner increasing transmission and the hospital was very well ventilated. For these case studies we generate predictions for the aerosol concentration, the dose inhaled, or the infection risk, depending on the data available, and we find good agreement. The model predictions also agree well with those made by CFD models, when they are available. More details are provided in the electronic supplementary material, §2.

### (a) Courtroom superspreader outbreak: comparison with data

**Figure 3.**
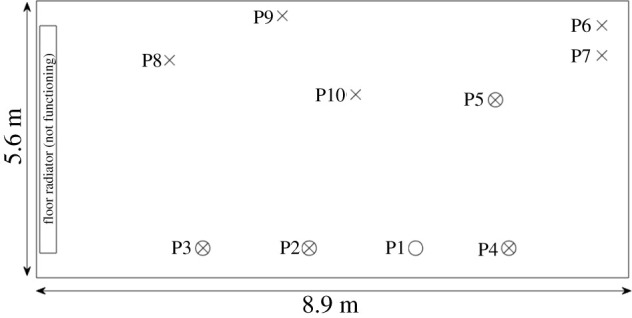
Schematic for the courtroom case study described in [30]. P1 is the infectious person, a circled X is an infected person, and X is an uninfected person.

We consider a courtroom in Vaud, Switzerland [30], that was poorly ventilated due to a breakdown in the mechanical ventilation system. Within days of a 3-h hearing in October 2020, five out of the 10 people present tested positive. Figure 3 shows the seating positions of the people in the courtroom. P1 was the infectious person. The infected people—P2, P3, P4, and P5—were seated within 3.5 m of P1, while people seated further away (P6–P9) were not infected. P10 also sat within 3.5 m but was in the room for only 34 min, as a witness.

We run our model for a three-hour 3-h event. As P1 was mostly silent according to the report of the hearing, we assume $R_{\text{total}}=8\;\mathrm{aerosols}/s$. Assuming a viral load $c_{v}=5\times 10^{9}$ (which is at the lower end of the viral loads required for aerosol transmission [68,72]) leads to μ=33% and hence R=2.5 aerosols/s. Taking also I=0.0069 corresponds to an emission rate of 62 quanta/h. This provides a spatially averaged probability of 39%, which matches well with four people infected by P1 out of nine. (Note that our quanta emission value is approximately half of the quanta emission rate of 130 quanta/h found by back-calculation in [30] using the Wells–Riley model.) In table 4 we present the dose inhaled by each person normalized by the dose inhaled by P2, who was closest to P1, as well as the infection risk. According to our model, P2–P5 were exposed to significantly more infectious aerosols than P6–P9 and, correspondingly, to a quite high infection risk compared with the not-infected individuals. This matches up with P2–P5 becoming infected. See electronic supplementary material, §2.1, for more details.

**Table 4.** Comparing the results of our model with data from the courtroom case study in [30] where five out of 10 people tested positive a few days after a 3-h hearing. P1 was the infectious person and infected P2–P5. P10 was in the room only for 34 min. We assume R=2.5 aerosols/s and I=0.0069, which is equivalent to 62 quanta/h. The dose was normalized by the dose inhaled by P2. We take parameter values as in table 2 and the electronic supplementary material, table S1, §2.1.

person	distance from infectious person	normalized dose	infection risk	infected? (Y/N)
P1	0 m	…	…	Y
P2	1.5 m	1.0	80.8%	Y
P4	1.5 m	1.0	83.1%	Y
P3	3 m	0.35	43.7%	Y
P5	3.3 m	0.23	35.3%	Y
P10	3.4 m	0.05	7.8%	N
P8	4.0 m	0.10	14.8%	N
P7	4.5 m	0.11	25.7%	N
P6	5.0 m	0.09	22.2%	N
P9	5.0 m	0.11	16.6%	N

### (b) Guangzhou restaurant superspreader outbreak: comparison with data and with CFD models

A superspreader outbreak occurred at a restaurant in Guangzhou, China, on 23 January 2020 [31]. In [17], they found that the airflow from the five air-conditioning units in the restaurant formed ‘recirculation zones’. In the recirculation zone where the infectious person was, the air-conditioning stream carried the infectious aerosols to the opposite window, then the stream bent downward and returned at a lower height, before finally the contaminated air rose and returned to the air-conditioning unit, repeating the cycle [17]. Seven to nine people out of 20 were infected in one of these recirculation zones [31], which corresponds to an infection rate of 35–45%.

**Figure 4.**
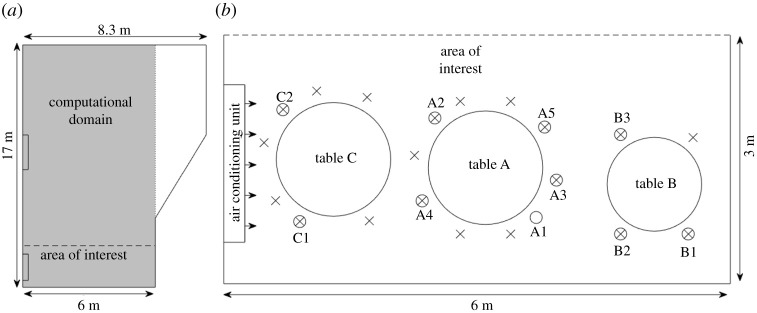
(*a*) The 6 × 17 m computational domain, shown in grey, over which we run our model as a simplified version of the Guangzhou restaurant [31]. (*b*) The area of interest contains the ‘recirculation zone’ in which seven to nine people were infected by A1, the infectious person. The circled X denotes an infected person and X denotes an uninfected person.

**Figure 5.**
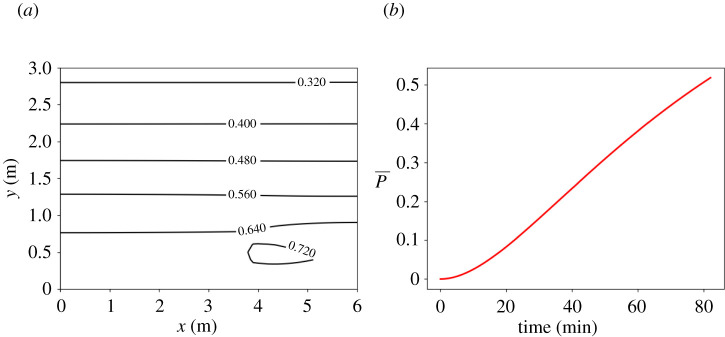
Superspreader outbreak in the Guangzhou restaurant in China [31]. (*a*) Infection risk after 82 min in the chosen recirculation zone. (*b*) Average infection risk, $\bar{P}$, versus time. We use (2.20) with l=6 m, w=17 m, h=3.14 m, $\lambda =0.77\;h^{-1}$, N=5 (five air-conditioning units). The infected person is at $\left(x_{0},y_{0})=(3.75,0.5\right)$ and we take R=10 (talking). Other parameter values taken as in table 2 and the electronic supplementary material, table S3. (Online version in colour.)

We simulate the concentration and infection risk using our model. We take as the computational domain a simplified rectangular domain 6 (l) × 17 (w) × 3.14 m (h)—see figure 4*a*. In figure 4*b*, we show in more detail the area of interest, which contains the recirculation zone in which the infectious person (A1) was sat, at Table A. We also show Tables B and C, which were also in this recirculation zone. Assuming that the infected person was talking without a mask, the emission rate R=5 aerosols/s, and I≈0.0069 (equivalent to 124 quanta/h). In figure 5*a*, we show the spatially varying infection risk and in figure 5*b* the average infection risk in the area of interest, as generated using our model, after 82 min. We have chosen 82 min since it is the time the infectious individual remained in the restaurant. The spatially averaged infection risk, $\bar{P}$, is 40% after an hour and over 50% after 82 min. Although 50% might be an overestimation of the infection risk, recall that we assumed the person was continuously talking, whereas s/he must have had periods when s/he was eating. Table 5 shows the average infection risk at Tables B, C, and E. Tables B and C in the area of interest have a significantly higher risk than Table E, which is an equal distances away from Table A but outside this area. Comparing also Tables B and C, which are similar distances away from Table A, the infection risk was lower at Table B since people stayed there 53 min versus 77 min at Table C. (See electronic supplementary material, §2.2, for more details.) Therefore, our model is in good agreement with the data. If the Wells–Riley model had been used, it would not have been able to identify the heightened risk in the area of interest. Furthermore, as the risk in the Wells–Riley model would have been averaged over the whole restaurant, back-calculation of the quanta emission rate would result in an extremely alarming figure. CFD models in [17,32,78] have also been used to study this superspreader case. Using our model, we find that in this region the average concentration after 1 h is five times higher than what the maximum concentration would have been without recirculation (see the electronic supplementary material, figure S5, §2, for more details); these results agree with [32]. With our model, we also determine the dose inhaled normalized by the dose inhaled by a person seated next to the infectious person; see table 5 and for more details, electronic supplementary material, §2.2. The result agrees well with [17].

**Table 5.** Normalized dose in the restaurant from [17], normalized by the dose inhaled by a person seated at Table A, adapted from table 3 from [17], and the normalized dose and average infection risk from our model, from (2.17) and (2.19) with l=6 m, w=17 m, h=3.14 m, $\lambda =0.77\;h^{-1}$, $V=431\;m^{3}$, N=5, $\left(x_{0},y_{0})=(3.75,0.5\right)$, and other parameter values as given in table 2 and the electronic supplementary material, table S3. Here, R=10, which corresponds to talking.

table	normalized dose in [17]	our normalized dose	our average infection risk
Table B	0.76	0.84	40.3%
Table C	0.89	0.85	57.7%
Table E	0.40	0.31	27.1%

### (c) Hospital ward: comparison with air-sampling data

**Figure 6.**
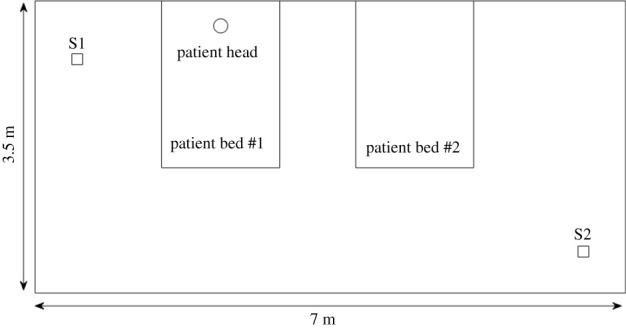
Schematic of the COVID-19 ward described in [33]. S1 and S2 represent the locations of the air samplers.

In [33], the concentration was sampled at two points in a COVID-19 hospital ward of area 7×3.5 m over 3 h. A single patient was present. We choose to compare with this case because it provides clear information regarding the dimensions of the room, the ventilation system, and the sampling positions. Figure 6 shows a schematic of the COVID-19 ward described in [33]. Sampling Position 1 (S1) was at the left wall, about 2 m from the head of the patient (the viral source), and sampling Position 2 (S2) was at the bottom-right corner of the room, about 4.8 m from the head of the patient. Ventilation was very good, with $\lambda =6\;h^{-1}$. Neither the activity of the patient nor the viral load were reported in [33]. The location-specific parameters are in the electronic supplementary material, table S5, §2, and other parameters are as in table 2.

Table 6 shows the comparison of our model with data from [33]. Our ratio of the concentrations at the two sampling locations is 1.74 while the experiment [33] gives 1.88, so there is good agreement. This shows the importance of employing a spatially dependent model like ours: the ratio arising from a Wells–Riley model would be equal to 1. More air-sampling data of aerosols carrying SARS-CoV-2 together with viral load measurements are required to accurately estimate Rμ in order to provide absolute values for the concentration (and the infection risk).

**Table 6.** Comparison of our predictions with air-sampling data from [33]. From (2.17) with parameter values as given in table 2 and the electronic supplementary material, table S5.

	concentration (virus copies/l)		
	S1	S2	S1/S2
[33] data	30	16	1.88
our model	$8.0\times 10^{-3}\;R\mu$	$4.6\times 10^{-3}\;R\mu$	1.74

## 5. Hypothetical case study: a classroom

**Figure 7.**
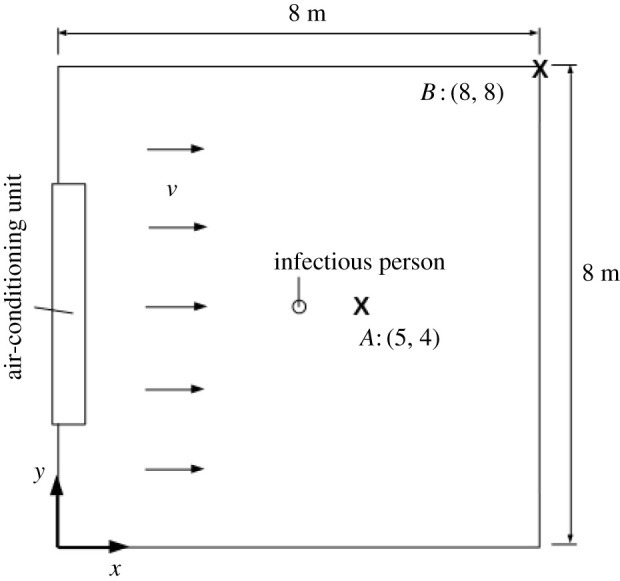
Schematic of the modelled classroom. One infectious person (viral source) is located at the centre of the room. Positions A:(5,4) and B:(8,8) are of particular interest and are studied in our analysis.

Countries have been debating the best practices for operating indoor spaces in schools and universities during the COVID-19 pandemic. Hence, we use our model to study the transmission of COVID-19 in an average classroom of dimensions 8 (l)×8 (w)×3 m(h)—see figure 7. For this classroom, we assume that the air-conditioning drives an airflow with velocity $v=0.15\;{m\;s}^{-1}$ [43]. All parameters associated with this location and their values are found in table 7.

**Table 7.** Location-dependent parameters and their values.

parameter	symbol	value	source
room length	l	8 m	
room width	w	8 m	
room height	h	3 m	
airflow speed	v	0.15 m s^−1^	[43]
room air exchange rate	λ	very poor ventilation: $0.12\;h^{-1}\approx 3.3\times 10^{-5}\;s^{-1}$	[79]
		poor ventilation: $0.72\;h^{-1}\approx 2\times 10^{-4}\;s^{-1}$	[79]
		ASHRAE recommended minimum ventilation for classrooms: $3\;h^{-1}\approx 8.3\times 10^{-4}\;s^{-1}$	[80,81]
		a ventilation better than the ASHRAE recommended minimum: $6\;h^{-1}\approx 1.7\times 10^{-3}\;s^{-1}$	[80]
eddy diffusion coefficient	K	very poor ventilation: $8.8\times 10^{-4}\;m^{2}\;s^{-1}$	[41]
		poor ventilation: $5.3\times 10^{-3}\;m^{2}\;s^{-1}$	[41]
		ASHRAE recommended minimum ventilation for classrooms: $2.2\times 10^{-2}\;m^{2}\;s^{-1}$	[41]
		ventilation better than the ASHRAE recommended minimum: $4.4\times 10^{-2}\;m^{2}\;s^{-1}$	[41]

Ventilation is increasingly considered a very important intervention against COVID-19 [82]. We will consider four different ventilation settings, as follows:

— Setting 1: Very poor ventilation,
— Setting 2: Poor ventilation,
— Setting 3: ASHRAE recommended minimum ventilation setting for classrooms [80,81],
— Setting 4: A ventilation setting better than the ASHRAE recommended minimum (that is equivalent to the ASHRAE recommended minimum for health care facilities [83]).

where ASHRAE is the American Society of Heating, Refrigeration and Air-Conditioning Engineers [81]. Each setting is characterized by a different value of λ, the air exchange rate, which increases with ventilation quality. The eddy diffusion coefficient, K, is determined by (2.8) for each λ assuming N=1 and its values are given in table 7.

### (a) The concentration of infectious aerosols

**Figure 8.**
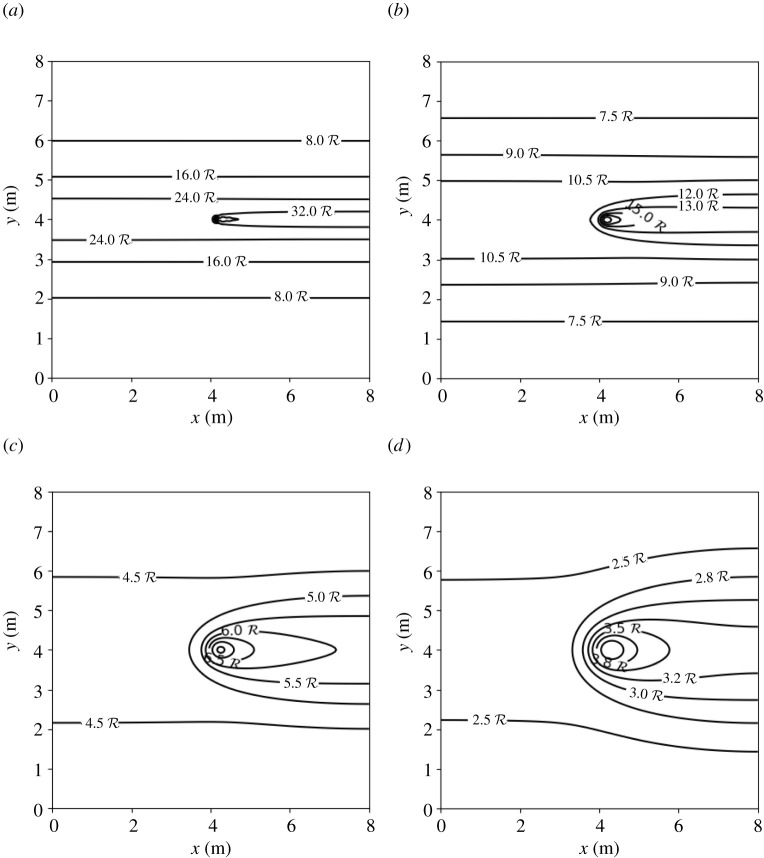
Concentration of SARS-CoV-2-carrying aerosols after 1 h in an 8×8×3 m room, determined from equation (2.17), with parameter values as given in tables 2 and 7, for four ventilation settings: (*a*) very poor ventilation, (*b*) poor ventilation, (*c*) ASHRAE recommended minimum ventilation for classrooms, and (*d*) ventilation better than the ASHRAE recommended minimum. Values for R for breathing and talking, with and without masks, are given in table 3.

Figure 8 shows, in a contour map, the concentration of the infectious aerosols in the classroom after 1 h, for the four ventilation settings. For all ventilation settings, the highest concentration is in the region directly downwind from the infectious person. The width of this region increases with the amount of ventilation, as it depends on the eddy diffusion coefficient, K, which is proportional to λ (see equation (2.8)). The next highest concentration in the room is found upwind. The concentration decreases as one travels away from the infectious person in the direction orthogonal to the airflow. Our results agree qualitatively with those obtained from air sampling in hospital wards in Wuhan, conducted in [84], which showed that virus-carrying aerosols were ‘mainly concentrated near and downstream from the patients’ and there was also an ‘exposure risk upstream’. Note that we express the concentration in terms of R so that the same plot can be used for breathing or talking, with and without a mask, or indeed other activities.

**Figure 9.**
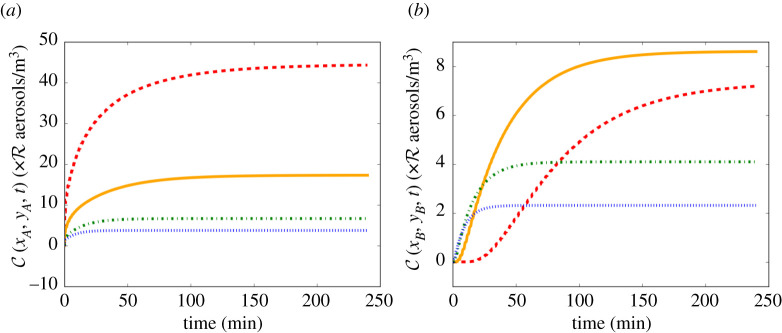
Concentration of SARS-CoV-2-carrying aerosols versus time in a case of very poor ventilation (red dashed), poor ventilation (orange solid), ASHRAE recommended minimum ventilation for classrooms (green dot-dashed), and ventilation better than the ASHRAE recommended minimum (blue dotted), from equation (2.17), with parameter values as given in tables 2 and 7. (*a*) Evaluated at Position A:(5,4). In this case, a power law of the form $C(x_{A},y_{A},t)\propto Rt^{\alpha }$ is obeyed, where α≈0.34; the trend deviates from this power law when t≈16 min and the presence of the walls begins to influence the solution. (*b*) Evaluated at Position B:(8,8). In this case, the influence from the walls is significant for all times and hence no scaling law is obeyed. Values for R for breathing and talking, with and without masks, are given in table 3. (Online version in colour.)

Figure 9 shows the concentration in the room versus time evaluated at Position A:(5,4) and Position B:(8,8) (figure 7). These two positions were chosen because, as seen in figure 8, the highest concentration is at Position *A* while maintaining 1 m social distancing from the infectious person and the lowest concentration is at Position *B*.

Figure 9 shows that the concentration increases initially, before reaching a steady state. For lower values of λ, more time is required to approach the steady-state concentration, as predicted also by the Gammaitoni–Nucci extension of the Wells–Riley model [22].

At point A, downwind from the source, figure 9*a* shows that the steady-state concentration is higher for lower values of λ, where there is less removal of infectious aerosols, as expected. Figure 9*a* also shows that we can obtain a power law of the form $C(x_{A},y_{A},t)\propto Rt^{\alpha }$, where α≈0.34 which is independent of the amount of ventilation; only the constant of proportionality depends on λ. The trend deviates from this power law (by more than 5%) when t≈16 min.

For positions that are outside the high-concentration regions, such as Position B in the corner, figure 9*b* shows that no scaling law is obeyed, due to the influence of the walls. Moreover, we see that the ASHRAE recommended ventilation settings 3 and 4 initially have higher concentrations before being overtaken by the poorer ventilation setting. This result occurs because the movement of the infectious aerosols in the direction orthogonal to the airflow is governed by the eddy diffusion coefficient, K, which increases with λ according to (2.8). Since the ASHRAE recommended ventilation settings have higher values of K, the infectious aerosols diffuse faster in the y-direction to begin with. However, given sufficient time, the infectious aerosols in the poorer ventilation settings will also reach the farthest points of the room and the concentrations there will eventually surpass those in the ASHRAE recommended ventilation settings. This result is a first indication that a small amount of ventilation could actually increase the risk of transmission compared with the case of very poor ventilation.

### (b) Probability of infection

**Figure 10.**
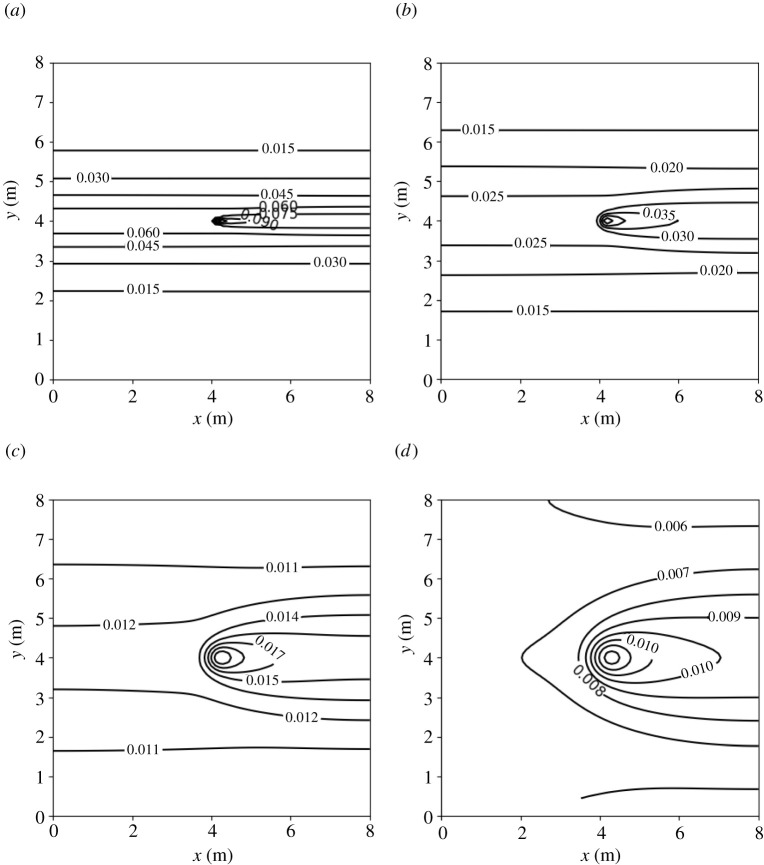
Probability of infection after 1 h due to an infectious person breathing at the centre of an 8×8×3 m room from (2.17) and (2.20), with parameter values as given in tables 2 and 7: (*a*) very poor ventilation, (*b*) poor ventilation, (*c*) ASHRAE recommended minimum ventilation for classrooms, and (*d*) ventilation better than the ASHRAE recommended minimum. Here, R=1, which corresponds to breathing.

Figure 10 shows the probability of infection for the four ventilation settings we considered in the 8×8×3 m room after an infectious person breathes for 1 h. The contours in figure 10 are similar in shape to the concentration contours in figure 8. This implies that the greatest risk of infection indoors is directly downwind from the infectious person and the risk decreases as we travel away from the source in a direction orthogonal to the airflow.

**Figure 11.**
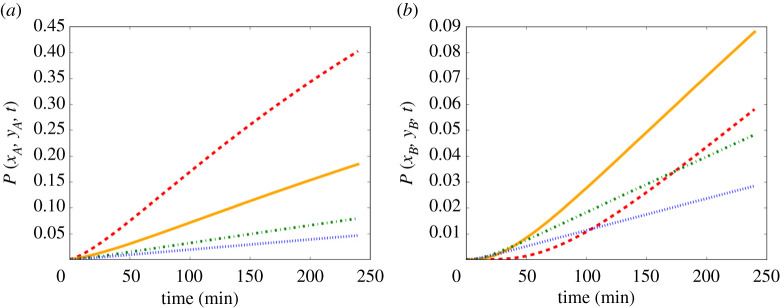
{Infection risk versus time in an 8×8×3 m classroom for an infectious person breathing in a case of very poor ventilation (red dashed), poor ventilation (orange solid), ASHRAE recommended minimum ventilation for classrooms (green dot-dashed), and ventilation better than the ASHRAE recommended minimum (blue dotted), using (2.17) and (2.20), with parameter values as given in tables 2 and 72. (*a*) Evaluated at Position *A*:(5,4). (*b*) Evaluated at Position *B*:(8,8). Here, R=1, which corresponds to breathing. (Online version in colour.)}

Figure 11 shows the probability of infection versus time evaluated at Position A and Position B in the case-study classroom for the case of an infectious person breathing. In figure 11*b*, we can see the effect of the concentration building more slowly at very poor ventilation that we saw in figure 9*b*, with the probability of infection growing very slowly initially then overtaking the two ASHRAE recommended ventilation settings 3 and 4. As Position A and Position B are the locations with the highest and lowest infection risks, respectively, in the room while maintaining social distancing of 1 m, by examining these two points we have a range of the risk in the room.

### (c) Average probability of infection

**Figure 12.**
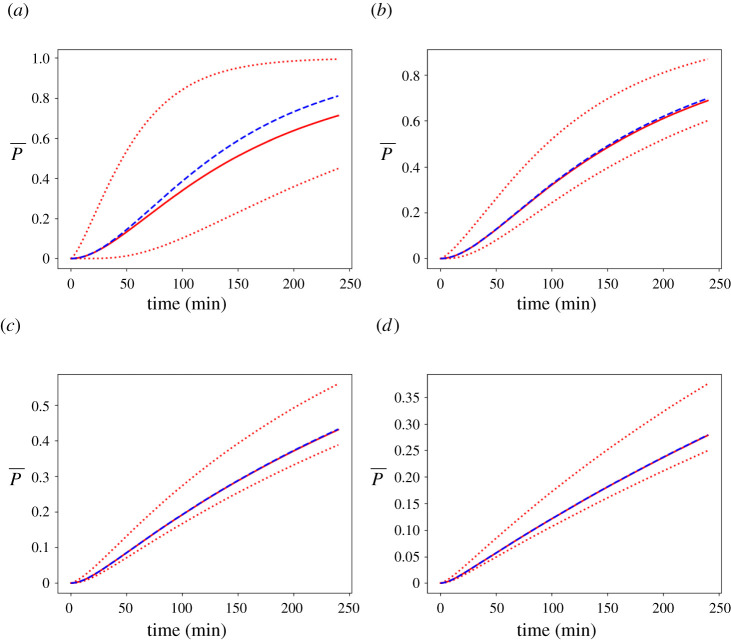
Comparison of the spatially averaged probability of infection $\bar{P}$ (red solid), and the infection risk at Positions *A* (upper red dashed) and *B* (lower red dashed), versus the probability of infection from the spatially averaged concentration (blue dashed) in an 8×8×3 m classroom with an infectious person talking, from (2.17) and (2.20), with parameter values from tables 2 and 7: (*a*) very poor ventilation, (*b*) poor ventilation, (*c*) ASHRAE recommended minimum ventilation for classrooms, and (*d*) ventilation better than the ASHRAE recommended minimum. Here, R=10, which corresponds to talking. (Online version in colour.)

In figure 12, we evaluate the spatially averaged probability of infection (2.20) and compare with the probability of infection derived by inserting the average concentration; the latter is according to the WMR assumption of the Wells–Riley model. We consider the infectious person talking. As figure 12*b*–*d* show, as ventilation improves, the spatially averaged probability is almost equal to the probability of the average concentration. However, for the very poor ventilation setting, figure 12*a* shows that the former is lower than the latter. This demonstrates that our spatially dependent model more accurately captures the infection risk in poorly ventilated environments compared with the Wells–Riley model.

### (d) Time to probable infection

**Figure 13.**
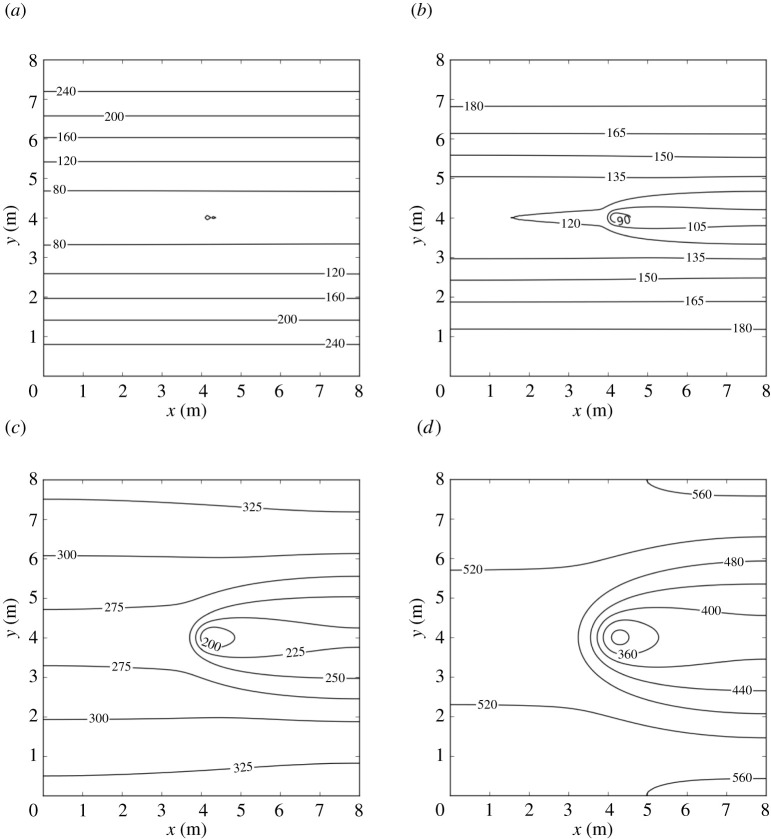
Time To Probable Infection (TTPI) due to an infectious person talking at the centre of an 8 × 8 × 3 m room from (2.17) and (2.20), with parameter values from 2 and 7: (*a*) very poor, ventilation, (*b*) poor ventilation, (*c*) ASHRAE recommended ventilation for classrooms, and (*d*) ventilation better than the ASHRAE recommended minimum. R=10 for talking.

Paving the way towards formulating policy recommendations, we can also use the model to calculate the Time To Probable Infection (TTPI)—the time required for the infection risk at a point in the room to reach 50%. We present in figure 13 the spatially dependent TTPI for the case of an infectious person talking in the 8×8×3 m classroom.

**Figure 14.**
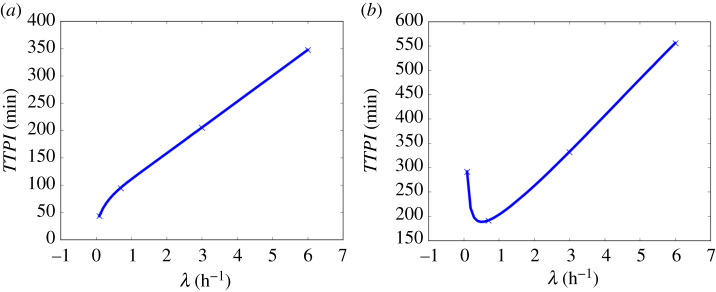
The TTPI versus the air exchange rate λ for an infectious person talking in the room; from (2.17) and (2.20): (*a*) at Position A:(5,4) and (*b*) at Position B:(8,8). All parameter values are given in tables 2 and 7. Here, R=10, which corresponds to talking. (Online version in colour.)

Figure 14 shows the relationship between the TTPI and λ at Positions A and B in our classroom case study, respectively. The TTPI monotonically increases with λ. We can extract the following values for the four ventilation settings. For very poor ventilation, TTPI≈50 min and for poor ventilation, TTPI≈100 min. For the two ASHRAE recommended ventilation settings, the TTPI exceeds 200 and 350 min, respectively. In figure 14*a*, we observe further evidence of the scaling-law behaviour at Position A that was identified in figure 9*a*, by now finding that the TTPI satisfies the relationship TTPI $\propto \lambda^{\gamma }$, where γ≈0.41 for $\lambda <1\;h^{-1}$, and a linear relationship, i.e. γ≈1, for $\lambda >1\;h^{-1}$. We note that as Position A is the most dangerous point in the room with the lowest TTPI (while maintaining 1 m social distancing), the TTPI at A could be used to determine a ‘safe occupancy time’ for the whole room.

At Position B, shown in figure 14*b*, at the top-right corner we see that the TTPI depends non-monotonically on λ with a TTPI attaining a minimum value (most dangerous point) for λ approximately equal to $0.6\;h^{-1}$. For values of λ lower than $0.6\;h^{-1}$ the TTPI *decreases* with λ; for values of $\lambda \;\text{higher than}\;0.6\;h^{-1}$ the TTPI again increases with λ. At first glance, this is perhaps counter-intuitive since it implies that more ventilation makes the room less safe, but it can be explained by the slower initial build-up of concentration for this range of λ (figure 9*b*). Crucially, this non-monotonic behaviour of the TTPI demonstrates that very low ventilation could increase the infection risk in some parts of the room away from the source, so a very important take-away message is that increasing the ventilation a little could actually be worse. This supports the initial observation made in figure 9. More significantly, as λ increases, the infection risks at both Positions *A* and *B* drop significantly and the TTPIs increase significantly—for example when we double λ from 3 to $6\;h^{-1}$ for *A*, we go from TTPI=200 to 370 min, and for *B*, we go from TTPI=325 to 570 min. We note that figures 13 and 14 could be generated very quickly for any location, parameters, and ventilation settings of interest.

**Figure 15.**
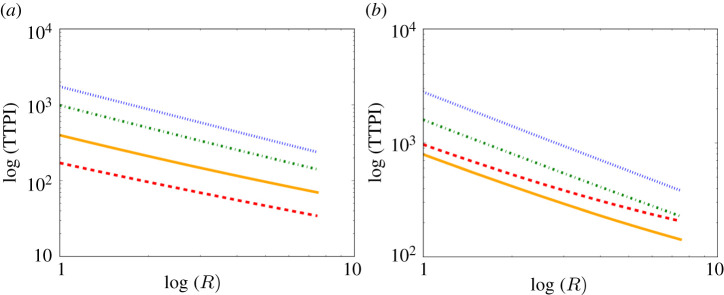
Log–log plot of the TTPI versus R in a case of very poor ventilation (red dashed), poor ventilation (orange solid), ASHRAE recommended minimum ventilation for classrooms (green dot-dashed), and ventilation better than the ASHRAE recommended minimum (blue dotted): (*a*) at Position A and (*b*) at Position B, obtained from (2.17) and (2.20). All parameter values are given in tables 2 and 7. (Online version in colour.)

Next, in order to quantify the dependence of the TTPI on the type of activity, we plot in figure 15 the TTPI versus R, the emission rate of infectious aerosols, for the four ventilation settings. At Position A there is a power law of the form $\mathrm{TTPI}\propto R^{\phi }$ with ϕ≈−0.79,−0.85,−0.96, and −0.99, respectively. At Position B, ϕ≈−0.75,−0.84,−0.96, and −0.99, respectively. As expected, the TTPI decreases as R increases, at both A and B. As ϕ decreases with increasing ventilation, the dependence of TTPI on R becomes weaker with increasing ventilation. In other words, the more aerosols generated per second the less safe the room is, but when ventilation increases sufficiently the type of activity matters less. We also note that at Position B, for the poorer ventilation settings, the effects of the weaker mixing are visible again. Furthermore, as ρ and I have the same influence in (2.20) as R, we could also determine analogous power laws of the forms $\mathrm{TTPI}\propto \rho^{\epsilon }$ or $\mathrm{TTPI}\propto I^{\nu }$.

**Figure 16.**
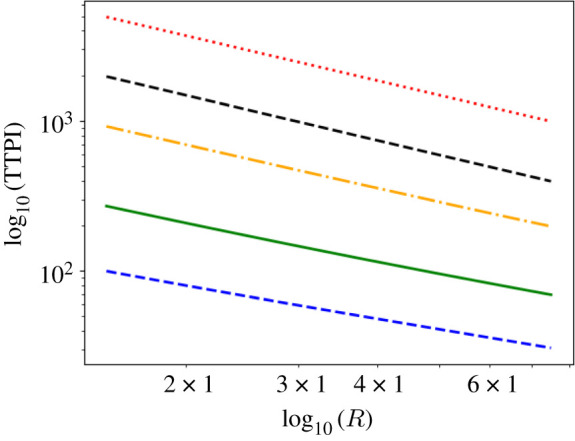
Log–log plot of the TTPI versus R, in rooms with poor ventilation, evaluated at 1 m downwind from the infectious person, from solving equations (2.17) and (2.20). The black dashed line represents an infinite room; the red dotted line represents a convention centre (68×105 m); the orange dot-dashed line represents an auditorium (15×30 m); the green solid line represents a classroom (8×8 m); and the blue dashed line represents a personal office (4×4 m). All other parameter values are given in table 2. (Online version in colour.)

In figure 16, we consider the TTPI at 1 m downwind in several other indoor spaces found in schools and universities: an office (4×4 m), an auditorium or lecture theatre (15×30 m), and a convention centre or indoor football field (68×105 m). We note that as the size of the room increases to infinity, ϕ→−1. Since ϕ decreases with increasing room size, this shows that the dependence of the TTPI on R becomes weaker with increasing room size.

## 6. Conclusion and future directions

In this paper, we have addressed the urgent need for a quick-to-run and efficient model that determines the spatially dependent concentration of airborne particles (aerosols) carrying the SARS-CoV-2 virus and also the corresponding probability of infection (infection risk). The model compares well with data from three real-life case studies in a courtroom [30], a restaurant [31], and a hospital ward [33], and also with CFD models (see §4). We have applied the model to predict the concentration and the infection risk in an average-sized classroom, and we have shown that the benefits of improving ventilation are significant. Our approach could be applied to any other room. We placed one infectious, asymptomatic, or presymptomatic person in the centre of the room who either breathes or talks, with or without a mask; this corresponds to four different emission rates for the viral aerosols (table 3). Note that we have taken the mask efficiency to be 50% but this number can be very easily altered (as well as all other parameters in the model).

The semi-analytic solution we derived in §2(c) allows for fast simulations; for example, it takes only 1 min of simulation to determine the concentration after 1 h of real time, on a standard laptop. Subsequently, using the spatially varying concentration (2.17) in the well-known exponential probability density function of the Wells–Riley model [20,29,52], we determine, for the first time, a spatially varying probability of infection (2.20).

The model relies on many parameters that are currently unknown for SARS-CoV-2 or which are known to vary over a wide range. Particularly uncertain parameters are the median infectious dose, and hence the conversion factor between dose of viral aerosols inhaled and infectious quantum, the viral load, and the length of time before the virus becomes biologically inactivated. We have used the best estimates for these parameters by consulting the literature and talking to scientists involved in experiments (see §3). However, our predictions must still be taken with caution. Our model provides an important contribution to the COVID-19 modelling field since it can be easily updated and rerun as and when more accurate estimates are obtained, or new estimates arise from new variants of the virus or from different types of activity, such as physical exercise (see §1 of electronic supplementary material).

In §5, we applied the model to a hypothetical average-sized classroom, with dimensions 8×8×3 m (figure 7). Our model shows that the concentration and the infection risk in the room are highest downwind from the infectious person (figure 8). As in the Wells–Riley model, the concentration in the room increases initially, then reaches a steady state (see figure 9). We consider the concentration at two indicative points: *A* (1 m downstream of the source, assuming social distancing); and *B* (top-right corner). The time to reach the steady state is shorter and the steady-state concentration is lower for larger values of ventilation λ at Position *A*. However, at Position *B* the highest concentration is attained in the poor ventilation setting. Moreover, the ASHRAE recommended ventilation settings 3 and 4 exhibit higher concentrations than the very poor ventilation for some time before the poor ventilation surpasses these values. At Position *A*, the concentration obeys a power-law dependence on time to begin with, since the spreading behaviour is unimpeded by obstacles. However, as time progresses and the effect of the walls comes into play, the power law breaks down. Analogously, no power law exists for Position *B*, since the influence of the walls is significant at all times due to their proximity to this location.

Another observation from figure 8 is that the second highest concentration level is found upwind of the source (on the same horizontal line). Our results agree qualitatively with air-sampling data from hospital wards in Wuhan [84], which showed that virus-carrying aerosols were ‘mainly concentrated near and downstream from the patients’ and there was also an ‘exposure risk upstream’.

In figure 10, we show the probability of infection for the four ventilation settings we consider (8 × 8 × 3 m classroom, infectious person breathing for 1 h). The contours in figure 10 are similar in shape to the concentration contours in figure 8. This implies that the greatest risk of infection indoors is directly downwind from the infectious person and the risk decreases as we travel away from the source in a direction orthogonal to the airflow. Figure 11 shows the probability of infection versus time evaluated at Positions A and B. In figure 11*b*, we see the effect of the concentration building more slowly for very poor ventilation (figure 9*b*), with the probability of infection growing very slowly initially then surpassing the two ASHRAE recommended ventilation settings 3 and 4. As Position A and Position B are the locations in the room with the highest and lowest concentrations, respectively, they provide us with the range of the concentration and the infection risk in the room.

In figure 12, we present a comparison of the spatially averaged probability of infection versus the probability of infection obtained from the average concentration arising from our model. The latter infection risk is that predicted by previous, spatially uniform Wells–Riley models. We consider an infectious person talking in the 8×8×3 m classroom. As figure 12*b*–*d* show, as ventilation improves, the average probability becomes almost equal to the probability derived from the average concentration. However, for the very poor ventilation setting, figure 12*a* shows that the spatially averaged probability is lower than the probability from the average concentration in the room. This figure suggests that the spatially averaged (WMR) approximations may overestimate the infection risk for poorer ventilation settings.

Paving the way for formulating policy-making recommendations, in §5(d) we used the spatially varying concentration to determine the TTPI as a function of ventilation λ. In the downwind region, 1 m away from the source (Position *A*), the TTPI increases with λ, exhibiting a scaling-law dependence on λ (figure 14*a*). Outside this high-concentration region (Position *B*), the TTPI, interestingly, exhibits a non-monotonic relationship with λ, first decreasing to a minimum TTPI and then increasing again with λ (figure 14*b*). This observation ties up with previous observations that improving ventilation a little may be worse than not doing anything. More significantly, figure 14 shows that increasing ventilation in a room can drastically reduce the infection risk and hence drastically increase the TTPI.

We have also identified a power-law relation between the TTPI and R, the emission rate of airborne infectious particles, (figure 15) that encapsulates the TTPI over a continuous range of R. Consequently, this can be used to quickly quantify the effect on the TTPI of the activity type for one person (for example, talking at low or high volume) or differences in the emission rate between persons (as some emit more viral particles than others for the same type of activity). We also note that this dependence of the TTPI on R becomes weaker with increasing room size (figure 16). Analogous power laws can be determined for the TTPI versus I, the conversion factor between the number of infectious aerosols and the infectious quantum in the probability density function (2.20); and for the TTPI versus ρ, the breathing rate of the susceptible person. These would allow us to quickly generate predictions for new variants, once the parameters are made available.

The model could be extended in several ways to account for additional questions of interest. Currently, we are working on including viral particle-size distributions, particularly size-dependent gravitational settling and emission rates that depend on the particle size. Also of particular practical interest is quantifying the effect on the infection risk when air purifiers are introduced in rooms; a stream of work motivated by our industrial partner Smart Separations Ltd. The model can be extended to study rooms with more complex geometries, air velocities, and ventilation systems. Ventilation could be modelled as a localized effect with the removal of the air occurring faster near the air vents and room openings [40].

In conclusion, our model can be implemented to assess the risk of airborne infection in rooms of different sizes when an infectious person is breathing or talking, with or without a mask. It can also be implemented to quantify the effectiveness of changing the ventilation available in the room. The modelling framework contains a series of parameters that may easily be adjusted in light of new guidelines on ventilation or confirmed information on the virus. Most importantly for the current COVID-19 pandemic, it can be implemented quickly on individual, widely available, and inexpensive computers.

## Data Availability

All scripts used in this study are openly accessible through https://github.com/zechlau14/Modelling-Airborne-Transmission. The data are provided in electronic supplementary material [85].

## Footnotes

1 This would not be a good assumption for coughing and sneezing.
